# EGR1 as a potential marker of prognosis in extranodal NK/T-cell lymphoma

**DOI:** 10.1038/s41598-021-89754-8

**Published:** 2021-05-14

**Authors:** Ji Yun Lee, Joo Hyun Kim, Heejin Bang, Junhun Cho, Young Hyeh Ko, Seok Jin Kim, Won Seog Kim

**Affiliations:** 1 Division of Hematology-Oncology, Department of Internal Medicine, Seoul National University Bundang Hospital, Seongnam, Korea; 2 Department of Health Sciences and Technology, Samsung Advanced Institute for Health Sciences and Technology, Samsung Medical Center, Sungkyunkwan University School of Medicine, Seoul, Korea; 3 Department of Pathology, Kangnam Sacred Heart Hospital, Hallym University College of Medicine, Seoul, Republic of Korea; 4 Department of Pathology and Translational Genomics, Samsung Medical Center, Sungkyunkwan University School of Medicine, Seoul, Korea; 5 Division of Hematology-Oncology, Department of Medicine, Samsung Medical Center, Sungkyunkwan University School of Medicine, 81 Irwon-ro, Gangnam-gu, Seoul, 06351 South Korea

**Keywords:** Haematological cancer, Tumour biomarkers

## Abstract

Extranodal natural killer T-cell lymphoma (ENKTL) is an aggressive malignancy with a dismal prognosis. In the present study, gene expression profiling was performed to provide more information on ENKTL molecular signature and offer a rationale for further investigation of prognostic markers in ENKTL. NanoString nCounter Analysis encompassing 133 target genes was used to compare gene expression levels of 43 ENKTL tumor samples. The majority of the patients were under 60 years of age (79.1%); 32 (74.4%) patients had nasal type ENKTL and 23 patients (53.5%) had intermediate/high risk ENKTL based on the prognostic index for natural killer cell lymphoma (PINK). The median follow-up was 15.9 months and the median overall survival (OS) was 16.1 months (95% CI 13.0–69.8). EGR1 upregulation was consistently identified in the localized stage with a low risk of prognostic index based on the PINK. Among the six significantly relevant genes for EGR1 expression, high expression levels of genes, including CD59, GAS1, CXCR7, and RAMP3, were associated with a good survival prognosis. The in vitro test showed EGR1 modulated the transcriptional activity of the target genes including CD59, GAS1, CXCR7, and RAMP3. Downregulation of EGR1 and its target genes significantly inhibited apoptosis and decreased chemosensitivity and attenuated radiation-induced apoptosis. The findings showed EGR1 may be a candidate for prognostic markers in ENKTL. Considerable additional characterization may be necessary to fully understand EGR1.

## Background

Extranodal natural killer T-cell lymphoma (ENKTL) is a subtype of mature T- and NK-cell lymphomas characterized by its association with Epstein–Barr virus (EBV) and extranodal involvement^1^. ENKTL is a rare aggressive malignancy with a unique geographical distribution encountered in East Asia and Central/South America^2^. The overall prognosis of ENKTL has improved in the era of non-anthracycline-based chemotherapy regimens^3,4^. However, patients with relapsed/refractory (R/R) ENKTL still have a poor prognosis and short overall survival (OS)^5^.

Recent studies of molecular biology and genetics have facilitated new avenues of investigation into ENKTL pathobiology and potential therapeutic targets. Based on gene expression profiling, several oncogenic pathways are activated, including Notch-1, Wnt, Janus kinase/signal transducers and activators of transcription (JAK/STAT), AKT, and nuclear factor-kappa B (NF-κB)^6,7^. Recently, next-generation sequencing (NGS) has identified frequent JAK3-activating mutations in ENKTL patients, indicating the JAK/STAT signaling pathway is a key molecular factor in the pathogenesis^8^. The prevalence of JAK3 mutations and STAT3 mutations in ENKTL was reported to range from 0–35% and 8–27%, respectively, in various studies^8–12^. In a preclinical study with tofacitinib, a pan-JAK inhibitor, effectively reduced tumor growth and metastatic spread of ENKTL, indicating JAK3 is a promising therapeutic target for ENKTL^8^. Programmed cell death ligand 1 (PD-L1) expression was reported in various studies to range from 56 to 93% in ENKTL, which has increased interest in using PD-1/PD-L1 inhibitors for ENKTL^10,13–15^. PD-1 blockade with pembrolizumab has shown promising activity in R/R ENKTL^16,17^. Whole-genome sequencing identified the PD-L1 mutation as a biomarker to select patients with ENKTL who are suitable for PD-1 blockade therapy^18^. Cho et al. showed immune subtyping of ENKTL to serve as a useful biomarker for checkpoint inhibitor-based immunotherapy^19^.

Despite progress in understanding ENKTL pathobiology, lymphomagenesis remains unresolved. In the present study, the molecular signature of ENKTL was characterized and potentially useful prognostic markers and/or therapeutic targets for treatment investigated.

## Results

### Distribution of patients

Patient demographics are summarized in Table 1. The majority of patients were under 60 years of age (79.1%), male (67.4%), and ECOG performance status 0 or 1 (81.4%). Among 43 patients, 31 (72.1%) were initially diagnosed with stage I or II disease and 35 (81.4%) had no distant lymph node involvement. Among the 32 patients who were tested for plasma EBV DNA using quantitative polymerase chain reaction (qPCR), 14 had a detectable viral DNA. Based on the PINK, 20 patients (46.5%) were classified into the low-risk group, 11 patients (25.6%) into the intermediate-risk group, and 12 patients (27.9%) into the high-risk group. Sixteen patients (37.2%; mainly with localized stage) received definitive concurrent chemoradiotherapy followed by chemotherapy.

**Table 1:** Patients characteristics (n = 43).

Characteristics	N (%)
**Age**	
≤ 60 years	34 (79.1%)
> 60 years	9 (20.9%)
**Sex**	
Male	29 (67.4%)
Female	14 (32.6%)
**Performance status**	
ECOG 0–1	35 (81.4%)
ECOG ≥ 2	8 (18.6%)
**Ann Arbor stage**	
I–II	31 (72.1%)
III–IV	12 (27.9%)
**Primary site**	
Nasal	32 (74.4%)
Non-nasal	11 (25.6%)
**Distant LN involvement**	
No	35 (81.4%)
Yes	8 (18.6%)
**EBV DNA**	
Undetectable	18 (41.9%)
Detectable	14 (32.6%)
Unknown	11 (25.6%)
**PINK**	
Low	20 (46.5%)
Intermediate	11 (25.6%)
High	12 (27.9%)
**PINK-E**	
Low	10 (23.3%)
Intermediate	9 (20.9%)
High	13 (30.2%)
Unknown	11 (25.6%)
**Primary treatment**	
Radiotherapy	3 (7.0%)
Chemotherapy	14 (32.6%)
Concurrent chemoradiotherapy followed by chemotherapy	16 (37.2%)
Chemotherapy followed by ASCT	3 (7.0%)
Chemotherapy followed by radiotherapy	4 (9.3%)
Not done	3 (7.0%)

*ECOG* Eastern Cooperative Oncology Group, *LN* lymph node, *EBV* Epstein–Barr virus, *PINK* prognostic index for natural killer cell lymphoma, *PINK-E* prognostic index for natural killer lymphoma–Epstein–Barr virus, *ASCT* autologous stem-cell transplantation.

### Survival analysis

**Figure 1:**
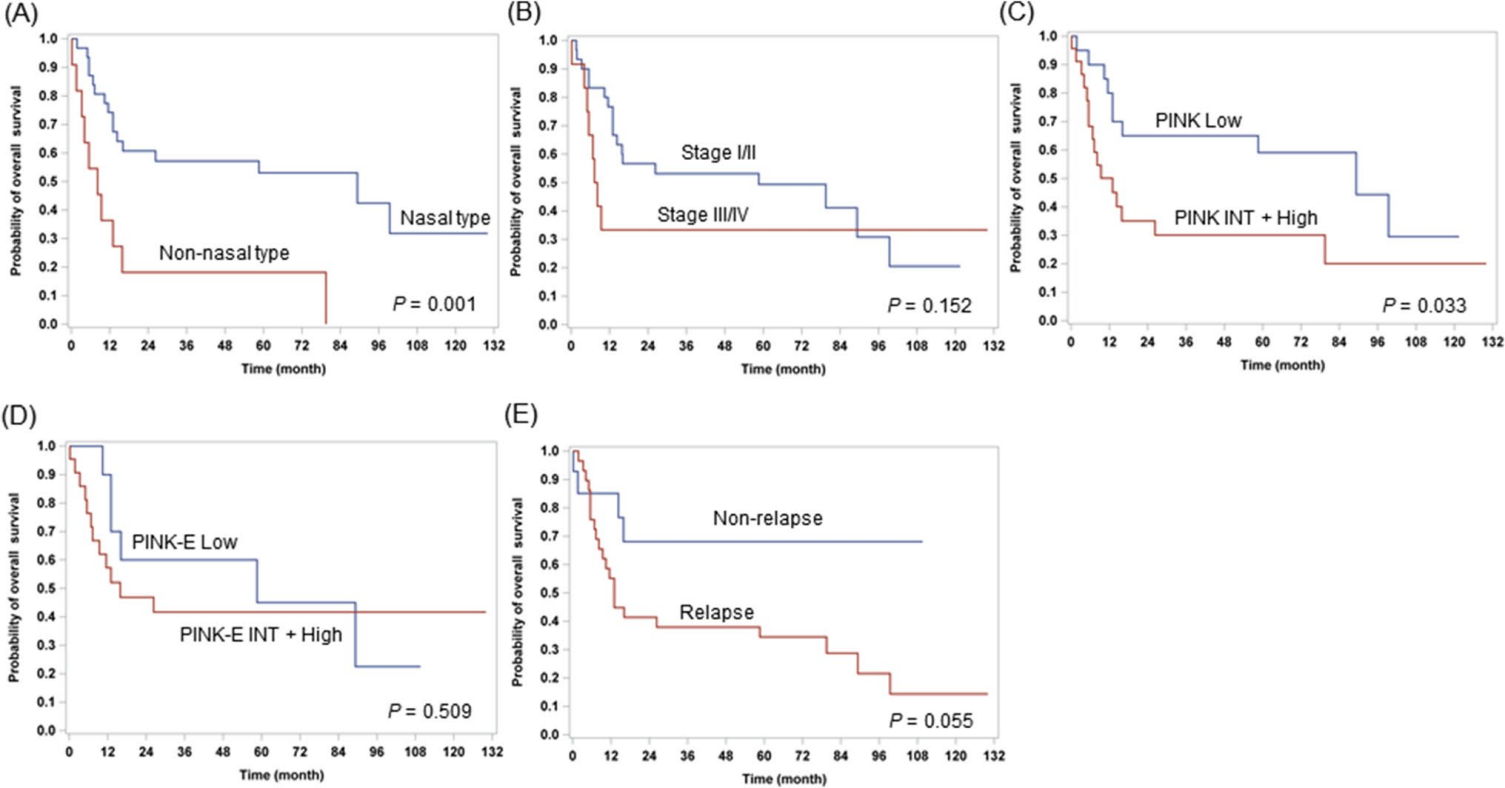
Kaplan–Meier survival curves for ENKTL patients based on primary site (**A**), Ann Arbor stage (**B**), PINK risk group (**C**), PINK-E risk group (**D**), and relapse (**F**). Data for PINK-E were obtained from the patients with available EBV data. ENKTL, extranodal natural killer T-cell lymphoma; PINK, prognostic index for natural killer lymphoma; PINK-E, prognostic index for natural killer lymphoma–Epstein–Barr virus; Epstein–Barr virus.

At the time of data analysis, disease in 29 patients (67.4%) had progressed after primary treatment and 26 (60.5%) had died, with a median follow-up of 15.9 months (range, 6.8–67.7 months). The median OS was 16.1 months (95% CI 13.0–69.8) and the 3-year OS was 47%. Kaplan–Meier analysis of the cohort showed increased mortality in non-nasal type (*P* = 0.001) and higher risk based on the PINK (*P* = 0.033; Fig. 1A, C). Significant statistical difference was not observed in OS based on the stage and PINK-E-derived risk due to the small sample size (Fig. 1B, D). The non-relapse group tended to have a better OS than the relapse group (Fig. 1E).

### Evaluation of prognostic effects of differentially expressed genes in ENKTL

**Figure 2:**
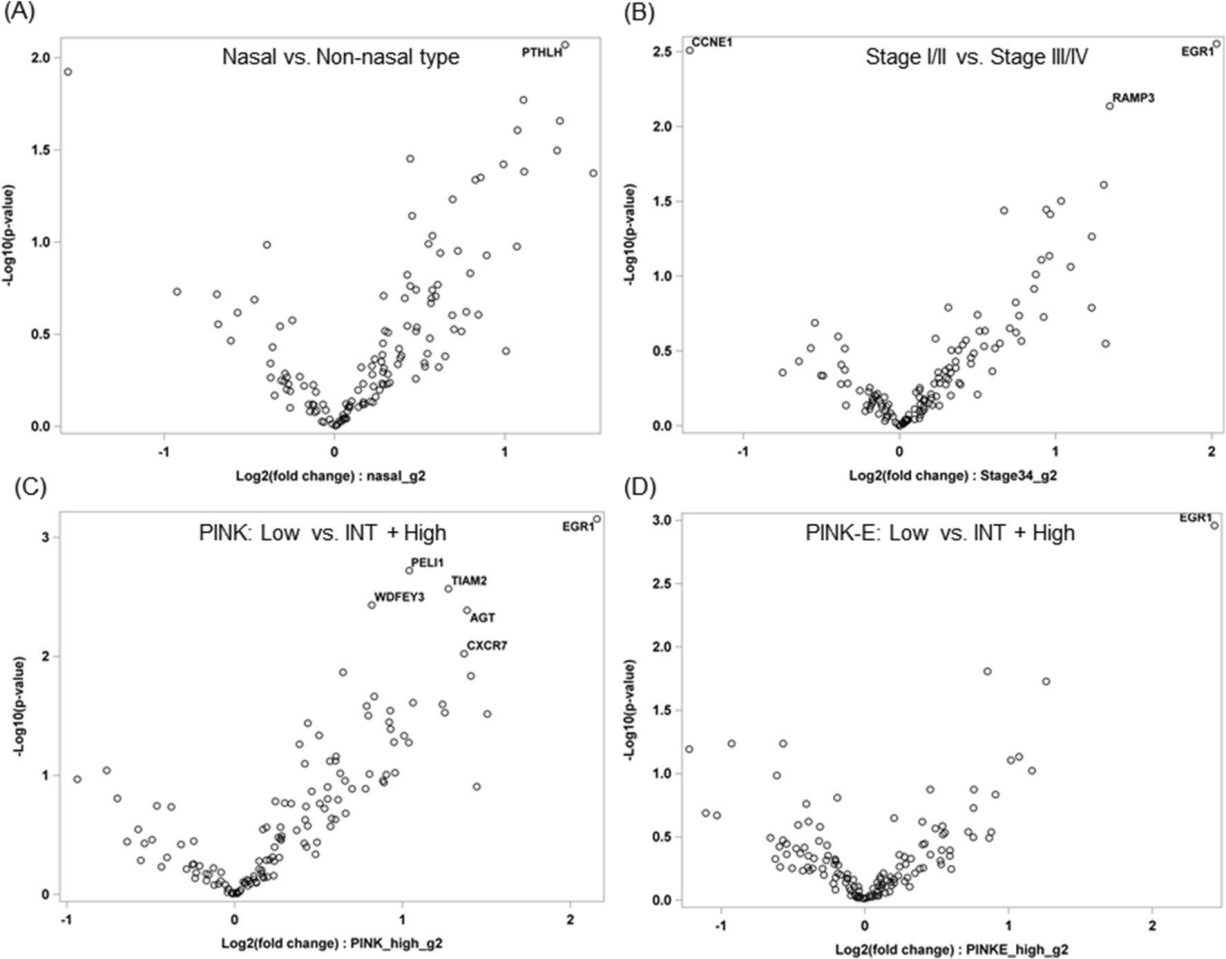
Volcano plots show the distribution of the fold changes in gene expression dependent on primary site, stage, and prognosis based on the PINK and PINK-E. Genes with absolute fold change ≥ 2 and the most statistically significant genes (*P* < 0.01) are listed. PINK, prognostic index for natural killer lymphoma; PINK-E, prognostic index for natural killer lymphoma-Epstein–Barr virus.

To identify patterns of gene expression based on the primary site, stage, PINK, and PINK-E risk groups in ENKTL, NanoString expression assay of 133 target genes was performed. PTHLH was upregulated in nasal type compared with non-nasal type (Fig. 2A). EGR1 and RAMP3 were upregulated in the localized stage and CCNE1 was upregulated in the advanced stage (Fig. 2B). Six genes, including EGR1, AGT, and CXCR7, were upregulated in the low-risk group based on the PINK (Fig. 2C). EGR1 was upregulated in the low-risk group based on the PINK-E (Fig. 2D).

EGR1 upregulation was consistently identified in the localized stage and low-risk group based on the PINK and PINK-E. Optimal cutoff value of EGR1 expression for predicting ENKTL prognosis based on the PINK was 95.3 (area under the curve, AUC = 0.7978) with a sensitivity of 78.3% and a specificity of 75.0% (Supplementary Fig. 1). Patients were divided into two groups based on low or high EGR1 expression levels. Analysis of the associations between clinical features and ERG1 expression (> 95.3 vs. ≤ 95.3) showed no correlation with nasal type or relapse, however, correlation was observed with low stage and good prognosis (Supplementary Table 1). Although not statistically significant, the group with high EGR1 expression showed a greater tendency to survive than the group with low EGR1 expression (*P* = 0.109; Supplementary Fig. 2). We next investigated whether there was a difference in EGR1 expression according to immune subtype. EGR1 expression was highest in immune-tolerant (IT) patients and tended to decrease in the order of immune evasion-A (IE-A), immune evasion-B (IE-B), and immune-silenced (IS) patients (*P* = 0.144; Supplementary Table 2).

**Figure 3:**
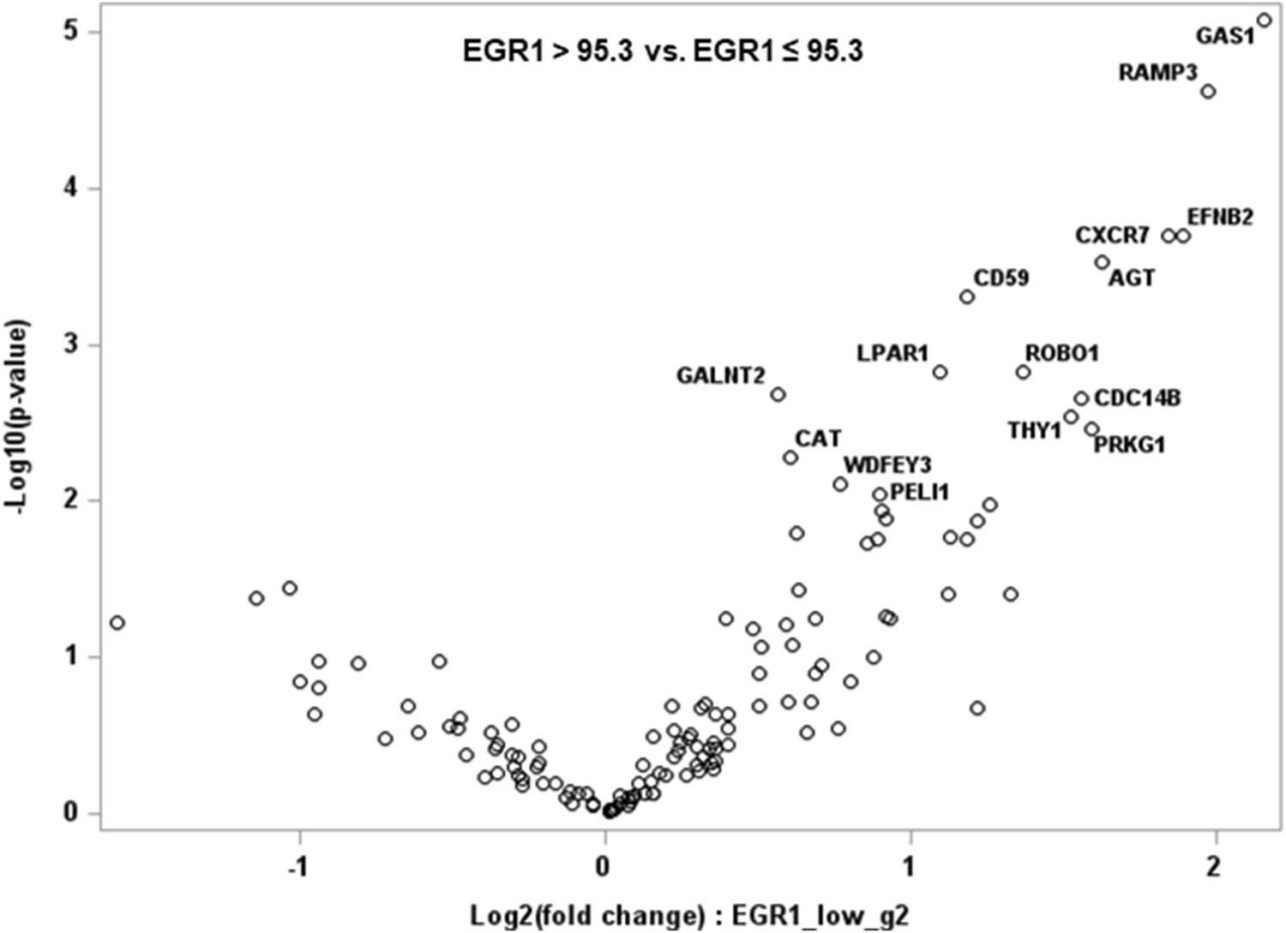
Volcano plots show the distribution of the fold changes in gene expression based on EGR1 expression. Genes with absolute fold change ≥ 2 and the most statistically significant genes (*P* < 0.01) are listed.

**Figure 4:**
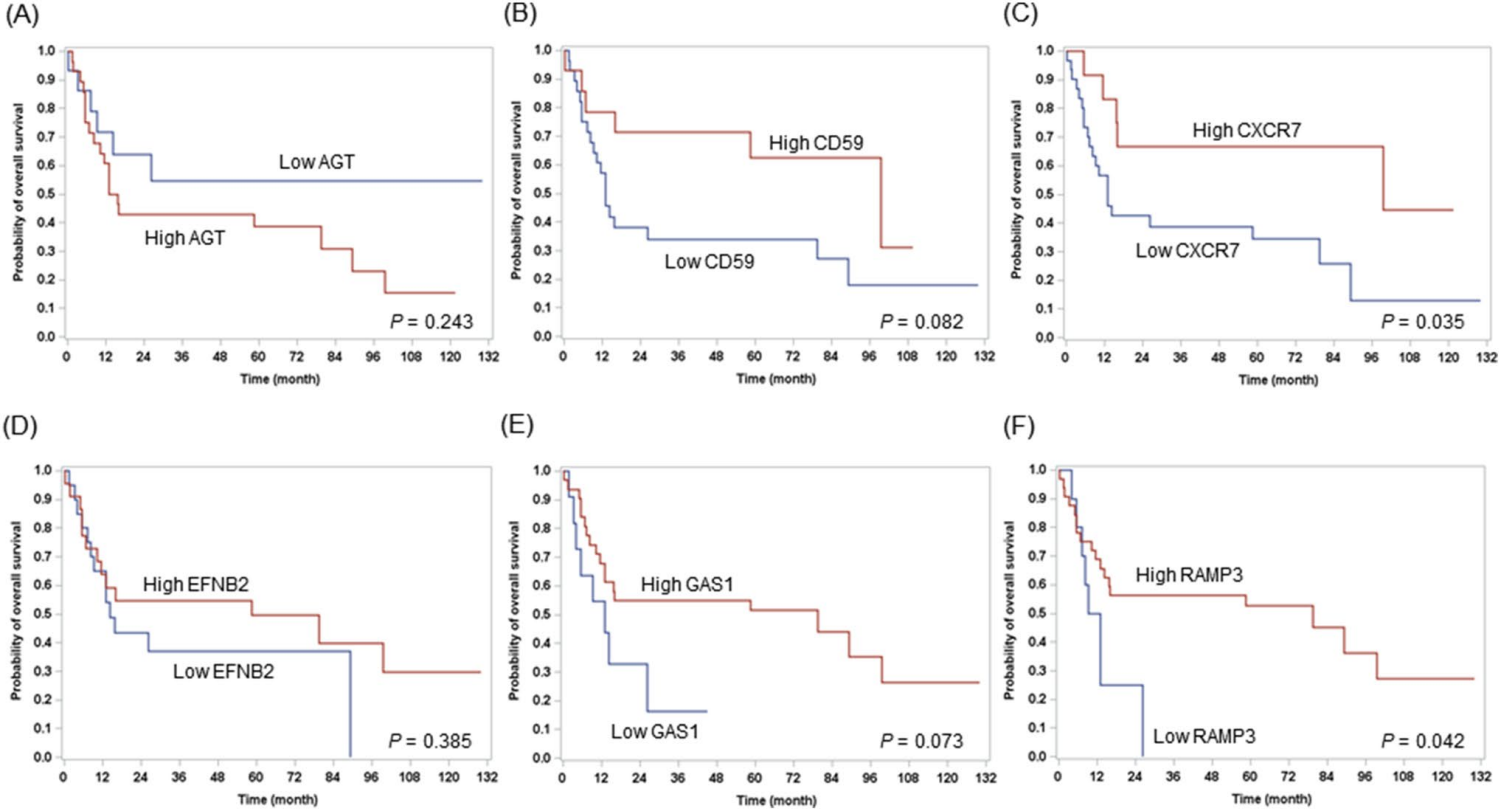
Kaplan–Meier survival curves for ENKTL patients based on gene expression of 6 genes including AGT (**A**), CD59 (**B**), CXCR7 (**C**), EFNB2 (**D**), GAS1 (**E**), and RAMP3 (**F**). ENKTL, extranodal natural killer T-cell lymphoma.

Figure 3 shows the distribution of the fold-changes in gene expression based on EGR1 expression (> 95.3 vs. ≤ 95.3) in ENKTL. Among the 15 significant genes (*P* < 0.01), the following 6 genes showed adjusted *P* < 0.05: AGT, CD59, CXCR7, EFNB2, GAS1, and RAMP3 (Supplementary Table 3). To further evaluate the prognostic effects of these genes in ENKTL**,** patients were dichotomized using the minimum *P* value of the log-rank test. Patients with high CD59 and GAS1 expression tended to have a better OS (Fig. 4B, E). High CXCR7 and RAMP3 expression were significantly associated with prolonged OS (Fig. 4C, F). There was no difference in survival rate according to the gene expression of AGT and EFNB2 (Fig. 4A, D).

### EGR1 as a regulator of multiple target genes including GAS1, CD59, CXCR7, and RAMP3

**Figure 5:**
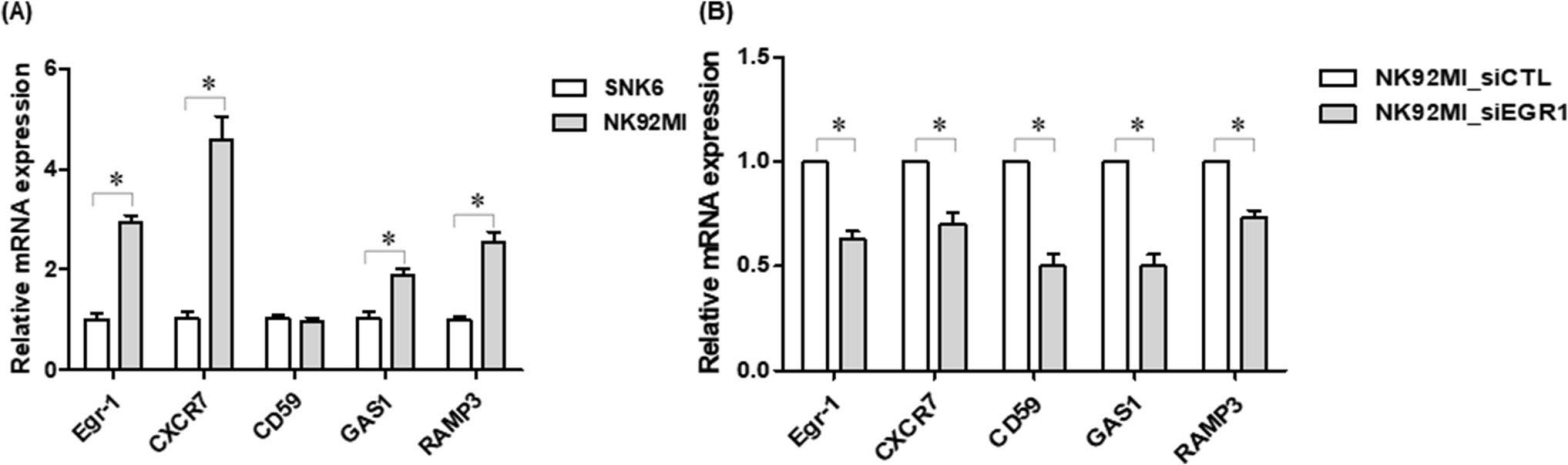
mRNA expression of EGR-1, CD59, CXCR7, GAS1 and RAMP3 was determined using qRT-PCR relative to levels in SNK6 using △△Ct method in NK/T lymphoma cell lines (**A**). NK92MI cells were transfected with siRNA control and siEGR-1. mRNA expression of EGR-1, CD59, CXCR7, GAS1 and RAMP3 was detected using qRT-PCR (**B**). *P* values were determined using Student’s t-test. *P < 0.05 versus siCTL. qRT-PCR, quantitative reverse transcription polymerase chain reaction; siRNA, small interfering RNA.

We examined the hypothesis that EGR1 regulates genes including GAS1, CD59, CXCR7, and RAMP3. We used two NK cell lines, SNK6 and NK92MI, for these studies; SNK6 cells are nasal NK/T cell lymphoma cells, are positive for CD56, and negative for CD3, CD4, CD8, CD19, and TCR. NK92MI cells are abundant in perforin and granzyme, suggesting cytotoxic effects. Therefore, NK92MI cells have become a critical NK cell line for preclinical research. The expression of these genes was higher in the NK92MI cell line compared with the SNK6 cell line (Fig. 5A), thus, the NK92MI cell line was used for further analysis. To determine whether EGR1 affected transcription of target genes including GAS1, CD59, CXCR7, and RAMP3, EGR1 was knocked down in the NK92MI cell lines. Figure 5B showed silencing of EGR1 significantly decreased CXCR7, CD59, GAS1, and RAMP3 expression, indicating EGR1 as a possible transcription factor that modulates the transcriptional activity of target genes including CD59, GAS1, CXCR7, and RAMP3.

**Figure 6:**
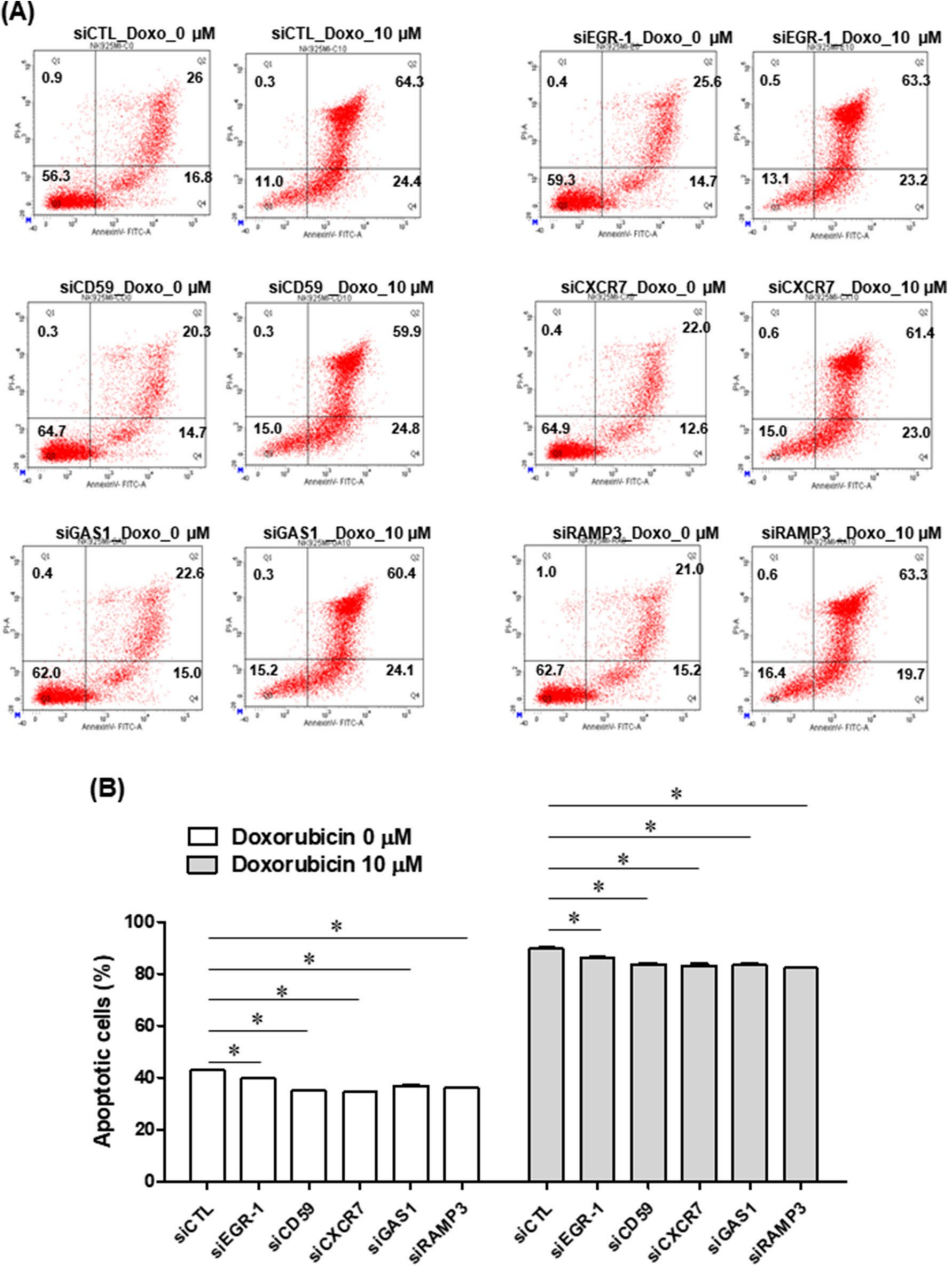
NK92MI cells were transfected with siRNA control, siEGR-1, siCD59, siCXCR7, siGAS1, and siRAMP3; 48 h later, doxorubicin (10 µM) was applied for 24 h (**A**). Dot plots are separated into four quadrants: Q1 (Annexin V-/PI + : cell death), Q2 (Annexin V + /PI + : late apoptotic cells), Q3 (Annexin V − /PI − : living cells), and Q4 (Annexin V + /PI- : early apoptotic cells). The percentage of apoptotic cells measured using flow cytometry (**B**). Values are presented as mean ± SEM (n = 3). *P* values were determined using Student’s t-test. *P < 0.05 versus siCTL siRNA, small interfering RNA.

**Figure 7:**
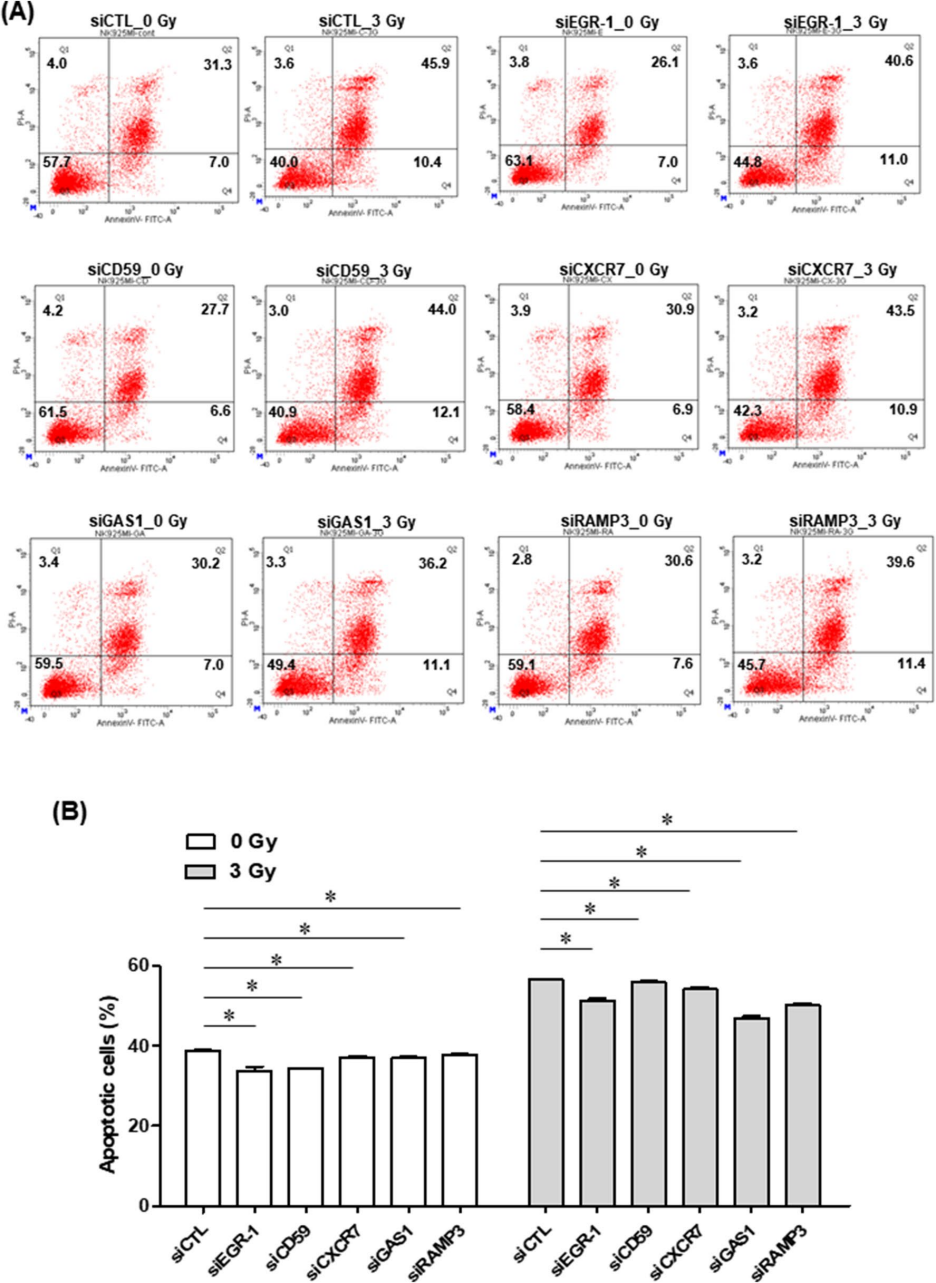
NK92MI cells were transfected with siRNA control, siEGR-1, siCD59, siCXCR7, siGAS1, and siRAMP3; 48 h later, gamma irradiation (3 Gy) was applied for 24 h, and apoptosis was measured (**A**). Dot plots are separated into four quadrants: Q1 (Annexin V − /PI + : cell death), Q2 (Annexin V + /PI + : late apoptotic cells), Q3 (Annexin V − /PI − : living cells), and Q4 (Annexin V + /PI − : early apoptotic cells). The percentage of apoptotic cells measured using flow cytometry (**B**). Values are presented as mean ± SEM (n = 3). *P* values were determined using Student’s t-test. *P < 0.05 versus siCTL siRNA, small interfering RNA.

Next, whether these genes could potentiate the apoptosis effects of chemotherapeutic agents and radiation-induced apoptosis against the NK92MI cell line was investigated. NK92MI cells were transfected with siRNA control, siEGR-1, siCD59, siCXCR7, siGAS1, and siRAMP3 and then exposed to doxorubicin (10 µM) for 24 h. The efficiency of knockdown through siRNA was confirmed through RT-PCR (Fig. 5B and Supplementary Fig. 3). Silencing of these genes induced a significantly lower percentage of apoptotic cells (*P* < 0.05) and rendered the NK92MI cells less sensitive to doxorubicin (*P* < 0.05; Fig. 6). These results were confirmed through reduction of cleaved PARP and caspase-3, which are apoptosis induction markers (Supplementary Fig. 4). The apoptotic rate was further examined in cells exposed to gamma irradiation (3 Gy). Knockdown of these genes significantly attenuated radiation-induced apoptosis (*P* < 0.05; Fig. 7).

## Discussion

Using the NanoString nCounter system, significant genes analyzed based on clinical factors, stage, and prognosis index using the PINK and PINK-E were identified.

In the current study, three important points were identified. First, EGR1 overexpression was associated with localized stage and low-risk group based on PINK and PINK-E in ENKTL. In addition, EGR1 overexpression appeared associated with prolonged survival, however, without statistical significance due to the small sample size. EGR1 is a zinc-finger transcription factor that binds and regulates cell growth, differentiation, and apoptosis^20^. EGR1 is a direct regulator of multiple tumor suppressors including TGFb1, PTEN, p53, and fibronectin^21^. EGR1 exhibits a biphasic expression behavior. In prostate, kidney, and stomach cancers, EGR1 stimulates the growth of tumor cells^22–24^, but is a tumor suppressor in esophageal cancer, breast cancer, and rhabdomyosarcoma^25–27^. The potential roles of EGR1 in the growth and proliferation of ENKTL have not been defined. Latent EBV infection plays a role in causing ENKTL, and EBV latent genes are important for malignant cell growth^28^. Among the EBV-encoded latent genes, LMP1 is a major EBV-encoded oncogene and activates NF-κB^29^. Microarray analysis by Kim et al^30^ revealed that EGR1 was a downstream cellular target of LMP1 via NF-κB in malignant T cells. Taken together, these results indicate latent EBV infection leads to induction of EGR1 in ENKTL.

Second, EGR1 expression promoted cell apoptosis by regulating target genes such as CD59, GAS1, CXCR7, and RAMP3 and was associated with good prognosis in ENKTL. CD59 is a key complement regulatory protein that restricts the formation of the membrane attack complex^31^. CD59 overexpression may assist malignant cells to escape immunologic surveillance and complement-mediated cytolysis, limiting the effects of complement-fixing monoclonal antibodies^32,33^. In several studies, GAS1 was reported a tumor suppressor and its downregulation associated with cancer progression and poor survival prognosis^34–36^. CXCR7 is a chemokine receptor that binds to the same ligand as CXCR4 and regulates the CXCR4-CXCL12 axis^37^. CXCR7 overexpression has been identified in several cancer types and found involved in the survival and growth of tumor cells^38^. CXCR7 overexpression was an independent prognostic marker associated with prolonged survival in diffuse large B-cell lymphoma patients^39^. The regulatory effects of RAMP3 varied significantly in different cancers. RAMP3 acts as a signal transducer of adrenomedullin, which stimulates cell proliferation, migration, and invasion in prostate and breast cancers^40,41^. However, increased RAMP3 expression was associated with better OS and reduced the negative effects of TP53 mutation on survival in recent studies^42,43^.

The third issue that, although the number of patients was too small to conclude statistical significance, EGR1 expression was highest in IT and lowest in IS. As a multi-functional transcription factor, EGR1 plays important roles in regulation of inflammation and the cellular immune responses to external stimuli^44^. Genes associated with reduced immune function, such as the transcriptional repressors EGR1 and BATF, are progressively upregulated during early tumorigenesis^45,46^. Cho et al. suggested that IT, IE-A, IE-B, and IS represent sequential stages of ENKTL disease progression^19^. Therefore, our results suggest that EGR1 is crucial in suppressing the immune response and, therefore, contributing to early tumorigenesis.

The present study is meaningful for the first time investigating the role of EGR1 in the pathogenesis of ENKTL, but there are several limitations. First, the relatively small sample size may provide an inaccurate representation of ENKTL. Second, the full analytical power could not be achieved due to the insufficient number of genes. Third, a functional study of target genes to investigate the roles of ENTKL pathogenesis was not performed. Further studies are being planned for survival analysis related to EGR1 expression based on immunohistochemistry of biopsy specimens from ENKTL patients and pathway enrichment analysis for investigating functional gene sets associated with ENKTL. Lastly, further studies are needed on the mechanism of EGR1 regulation of T cell state to contribute to tumor cell immune evasion. EGR1 and the pathways that control its activity may provide new opportunities for immunotherapy in ENKTL.

In summary, the results indicate that EGR1 may represent a useful prognostic marker in ENKTL Identification of patients with low EGR1 may distinguish patients at high risk of disease and who have tumors resistant to therapy secondary to loss of pathways such as GAS1, CD59, CXCR7, and RAMP3. The reintroduction of the EGR1 gene might be a promising therapeutic intervention that will improve the efficacy of treatment for ENKTL.

## Methods

### Patient population

ENKTL tumors (n = 43) were obtained from the Samsung Medical Center. Baseline patient characteristics collected for analysis included age, sex, Eastern Cooperative Oncology Group (ECOG) performance status, Ann Arbor stage, involvement of the nasal cavity or nasopharynx, and regional or distant lymph nodes. Nasal and non-nasal types were defined based on involvement of the nasal area^47^. A prognostic index for ENKTL was assessed as low-risk, intermediate-risk, or high-risk groups based on the prognostic index for natural killer cell lymphoma (PINK)^48^. PINK-Epstein–Barr virus (PINK-E) was obtained for patients with data for peripheral blood EBV DNA status. Any detectable concentration of EBV DNA was defined as positive. Of the 43 total patients, 30 had information on immune subtyping to classify ENKTL patients into four tumor immune microenvironment subgroups of IT, IE-A, IE-B, and IS^19^.

All patients provided written informed consent for the use of archival tissues with retrospective clinical data. This study was performed in accordance with the Declaration of Helsinki and approved by the Institutional Review Board of Samsung Medical Center.

### RNA extraction

All available hematoxylin and eosin (H&E)-stained slide from archival formalin-fixed, paraffin-embedded (FFPE) tissues were reviewed by two pathologists (H.B. and Y.H.K.). Total RNA was extracted from 2 to 4 sections of 4-μm-thick sections from whole FFPE tumor tissues using the High Pure RNA Paraffin kit (Roche Diagnostic, Mannheim, Germany). The RNA was quantitated using UV spectroscopy (Nanodrop Technology, Wilmington, DE, USA).

### NanoString nCounter assay using a probe for 137 genes

NanoString-based multigene assay was performed according to the published literature^49^. Briefly, 200 ng total RNA was used to determine gene expression levels utilizing NanoString technology (NanoString Technologies, Seattle, WA, USA). An nCounter CodeSet (NanoString Technologies) containing a biotinylated capture probe for 133 target genes and 4 housekeeping genes (ACTB, B2M, G6PD, and GAPD; Supplementary Table 4) was used for gene expression analyses. The data were normalized to the mean expression levels of internal reference genes with a cutoff value of 20. Standard quality control was performed with nSolver Analysis Software (NanoString Technologies) with flagging of any sample with a total of the positive spike-in controls outside of 0.3–3 times the geometric mean of the total positive spike-in for that cartridge. Two samples in these experiments were flagged. The probe counts were then normalized using the geometric mean of the 4 housekeeping genes and log2 transformed for further analysis.

### Cell lines

SNK6 was kindly provided by Dr. Y. K. J (Seoul National University Hospital, Seoul, Korea) and NK92MI were purchased from American Type Culture Collection (Rockville, MD, USA). SNK6 was cultured in RPMI-1640 medium supplemented with 10% heat-inactivated fetal bovine serum (FBS) and 500 U/mL of interleukin-2. NK92MI was cultured in MEM-α medium supplemented with 20% heat-inactivated FBS. Penicillin and streptomycin (Gibco-BRL, Grand Island, NY, USA) were added to the media and cells were incubated in a humidified 5% CO_2_ atmosphere. Small interfering RNAs (siRNAs) were purchased from Bioneer (Daejeon, South Korea). Cells were transiently transfected using Lipofectamine RNAiMAX (Invitrogen, Carlsbad, CA, USA). Doxorubicin was purchased from Apexbio (Boston, MA, USA).

### Quantitative reverse transcription polymerase chain reaction (qRT-PCR)

Total cellular RNA was isolated using a Qiagen RNA extraction kit (Qiagen, Valencia, CA, USA), according to the manufacturer’s instructions. For reverse transcription, 1 μg of RNA was treated with RNase-free DNase, and cDNA obtained using an Omniscript RT kit (Qiagen) according to the manufacturer’s protocol. The generated cDNA was amplified using primers specific for EGR-1, CD59, CXCR7, GAS1, and RAMP3 (Supplementary Table 5). GAPDH was amplified as the reference gene.

### Apoptosis assay

Apoptosis was detected using an Annexin V-fluorescein isothiocyanate (FITC) Kit (BD Biosciences, San Jose, CA, USA) and a BD FACS ARIA III flow cytometry system (BD Biosciences) according to the manufacturer’s protocol. Cells were exposed to doxorubicin or gamma irradiation, then harvested and processed according to the manufacturer’s instructions.

### Western blot analysis

The antibodies employed were those specific for PARP, cleaved caspase-3, HRP-conjugated horse anti-mouse IgG (all from Cell Signaling), and HRP-conjugated goat anti-rabbit IgG (from Santa Cruz Biotechnology). β-Actin (from Sigma) was detected as a loading control. All primary antibodies were diluted 1:1000, and secondary antibodies were diluted 1:3000.

### Statistical analysis

A two-sample t-test was used to determine differential expression of genes between groups. The t-test *P* value of each gene was calculated based on 10,000 permutations and the adjusted *P* value was calculated using the single-step procedure to control the family-wise error rate (FWER)^50^. The Kaplan–Meier method was used to estimate OS rates and the log-rank test was used to compare survival distributions between the groups. The multivariate Cox regression analysis was used to identify risk factors associated with significant genes. The optimal cutoff was selected as the point with the most significant log-rank *P* value for all possible cutoff points. Statistical analysis was performed using R 3.0.2 (Vienna, Austria; http://www.R-project.org/) and SAS 9.4 (SAS Institute, Cary, NC, USA).

### Remark guidelines

When reporting this study, we adhered to the guidelines of an important methodological paper from 2005 entitled “Reporting recommendations for tumor marker prognostic studies (REMARK guidelines)”^51,52^.

## Supplementary Information

Supplementary Information.
