# Supplementary material for: EGR1 as a potential marker of prognosis in extranodal NK/T-cell lymphoma
Source: Sci Rep. 2021 May 14;11:10342. doi: 10.1038/s41598-021-89754-8 (PMC8121831; doi:10.1038/s41598-021-89754-8)

**Original article**

**EGR1 as a Potential Marker of Prognosis in Extranodal NK/T-cell Lymphoma**

Ji Yun Lee^1*^, Joo Hyun Kim ^2*^, Heejin Bang^3^, Junhun Cho^4^, Young Hyeh Ko^4^, [Seok Jin Kim](javascript:;)^2^, [Won Seog Kim](javascript:;)^2^

^1^Division of Hematology-Oncology, Department of Internal Medicine, Seoul National University Bundang Hospital, Seongnam, Korea

^2^Department of Health Sciences and Technology, Samsung Advanced Institute for Health Sciences and Technology, Samsung Medical Center, Sungkyunkwan University School of Medicine, Seoul, Korea

^3^Department of Pathology, Kangnam Sacred Heart Hospital, Hallym University College of Medicine, Seoul, Republic of Korea

^4^Department of Pathology and translational genomics, Samsung Medical Center, Sungkyunkwan University School of Medicine, Seoul, Korea

^5^Division of Hematology-Oncology, Samsung Medical Center, Sungkyunkwan University School of Medicine, Seoul, Korea

**Supplementary table 1. Association between EGR1 expression and patient’s characteristics**

|  | EGR1 > 95.3, n (%) | EGR1 ≤ 95.3, n (%) | *P*-value |
| --- | --- | --- | --- |
| Primary site |  |  |  |
| Nasal | 17 (85.0) | 15 (65.2) | 0.1381 |
| Non-nasal | 3 (15.0) | 8 (34.8) |  |
| Stage |  |  |  |
| I-II | 19 (95.0) | 12 (52.2) | 0.0018 |
| III-IV | 1 (5.0) | 11 (47.8) |  |
| PINK |  |  |  |
| Low | 15 (75.0) | 5 (21.7) | 0.0005 |
| Intermediate/High | 5 (25.0) | 18 (18.3) |  |
| PINK-E |  |  |  |
| Low | 8 (61.5) | 2 (10.5) | 0.0051 |
| Intermediate /High | 5 (35.5) | 17 (89.5) |  |
| Relapse |  |  |  |
| No | 7 (35.0) | 7 (30.4) | 0.7500 |
| Yes | 13 (65.0) | 16 (69.6) |  |

PINK, prognostic index for natural killer cell lymphoma; PINK-E, prognostic index for natural killer lymphoma–Epstein-Barr virus

**Supplementary table 2. EGR1 expression according to the immune subtyping**

| Immune subtype | IT | IE-A | IE-B | IS | *P*-value |
| --- | --- | --- | --- | --- | --- |
|  | (n = 3) | (n = 16) | (n = 9) | (n = 2) |  |
| EGR1 | 395.1 [272.6;546.3] | 185.1  [53.1;323.1] | 90.8  [42.0;178.3] | 41.6  [19.2;64.0] | 0.144 |

IT, immune tolerance; IE-A, immune evasion-A; IE-B, immune evasion-B; IS, immune silenced (IS).

**Supplementary table 3.** Genes differentially expressed between EGR1 high expression (> 95.3) and EGR1 low expression ≤ 95.3

|  | EGR1 expression | | *P*-value | Adjusted *P*-value |
| --- | --- | --- | --- | --- |
|  | > 95.3 | ≤ 95.3 |  |  |
| CAT | 7.3440 | 6.7399 | 0.0052 | 0.3072 |
| CDC14B | 5.1979 | 3.6403 | 0.0022 | 0.1612 |
| GALNT2 | 7.3430 | 6.7764 | 0.0021 | 0.1968 |
| LPAR1 | 4.9832 | 3.8871 | 0.0015 | 0.1035 |
| PELI1 | 7.3144 | 6.4157 | 0.0089 | 0.5208 |
| PRKG1 | 3.3276 | 1.7344 | 0.0035 | 0.2430 |
| ROBO1 | 5.7998 | 4.4335 | 0.0015 | 0.1705 |
| THY1 | 7.1319 | 5.6051 | 0.0029 | 0.2852 |
| WDFEY3 | 5.4375 | 4.6670 | 0.0077 | 0.3830 |
| AGT | 2.4056 | 0.7759 | 0.0003 | 0.0327 |
| CD59 | 9.2349 | 8.0465 | 0.0005 | 0.0320 |
| CXCR7 | 4.8212 | 2.9727 | 0.0002 | 0.0159 |
| EFNB2 | 5.1930 | 3.3002 | 0.0002 | 0.0204 |
| GAS1 | 5.4216 | 3.2634 | 0.0000 | 0.0027 |
| RAMP3 | 5.6366 | 3.6654 | 0.0000 | 0.0003 |

The six genes marked in red showed adjusted P < 0.05.

**Supplementary table 4. 133 nCounter genes list**

| **No.** | **Genes** | **No.** | **Genes** | **No.** | **Genes** | **No.** | **Genes** |
| --- | --- | --- | --- | --- | --- | --- | --- |
| **1** | ADRB2 | **35** | CSF2 | **69** | KIR2DL1 | **103** | SH2D1B |
| **2** | AGT | **36** | CTLA4 | **70** | KIR2DL2 | **104** | SIRPA |
| **3** | ALK | **37** | CTSB | **71** | KIR2DL4 | **105** | SLC16A3 |
| **4** | ALS2 | **38** | CTSC | **72** | KIR2DS1 | **106** | SLC19A1 |
| **5** | ANK2 | **39** | CTSS | **73** | KIR2DS2 | **107** | SLC19A2 |
| **6** | ANXA3 | **40** | CXCR3 | **74** | KIR2DS5 | **108** | SLC7A7 |
| **7** | ARHGEF10 | **41** | CXCR4 | **75** | KLRC2 | **109** | SMARCA2 |
| **8** | ATP6V0D1 | **42** | CXCR7 | **76** | KLRD1 | **110** | SMOX |
| **9** | AXL | **43** | CYBB | **77** | LILRB1 | **111** | SNAP91 |
| **10** | BATF3 | **44** | CYP26A1 | **78** | LPAR1 | **112** | SOD2 |
| **11** | BTBD11 | **45** | DMRT1 | **79** | MSH6 | **113** | SOX8 |
| **12** | CAT | **46** | DNER | **80** | MYCN | **114** | SPAST |
| **13** | CCNA1 | **47** | EFNB2 | **81** | NCAM1 | **115** | STAT1 |
| **14** | CCNE1 | **48** | EGR1 | **82** | NKX2_1 | **116** | STK3 |
| **15** | CCR1 | **49** | FABP3 | **83** | NLRP7 | **117** | TBX21 |
| **16** | CCR10 | **50** | FASLG | **84** | NOTCH1 | **118** | TCN2 |
| **17** | CCR11 | **51** | FGF18 | **85** | NR1H3 | **119** | THY1 |
| **18** | CCR2 | **52** | FNTB | **86** | PCOLCE2 | **120** | TIAM2 |
| **19** | CCR3 | **53** | FPR1 | **87** | PDE4DIP | **121** | TMEM158 |
| **20** | CCR4 | **54** | FTL | **88** | PDXK | **122** | TMOD1 |
| **21** | CCR5 | **55** | GALNT2 | **89** | PELI1 | **123** | TNFRSF8 |
| **22** | CCR6 | **56** | GAS1 | **90** | PERP | **124** | TUBB2B |
| **23** | CCR7 | **57** | GATA3 | **91** | PITPNA | **125** | TUBB6 |
| **24** | CCR8 | **58** | GUCA2A | **92** | PLSCR1 | **126** | TYR |
| **25** | CCR9 | **59** | HCK | **93** | PRDX3 | **127** | UBE2L6 |
| **26** | CD244 | **60** | HPSE | **94** | PRKCB | **128** | WARS |
| **27** | CD28 | **61** | HRASLS | **95** | PRKG1 | **129** | WDFEY3 |
| **28** | CD40 | **62** | HS6ST2 | **96** | PSAP | **130** | WNK1 |
| **29** | CD59 | **63** | IFI30 | **97** | PTHLH | **131** | WNT7B |
| **30** | CDC14B | **64** | IFNG | **98** | RAMP3 | **132** | ZBTB17 |
| **31** | CHI3L1 | **65** | IL13RA1 | **99** | REL_1 | **133** | ZFYVE27 |
| **32** | CLTC | **66** | IL1RAP | **100** | ROBO1 |  |  |
| **33** | COL6A1 | **67** | JAK2 | **101** | S1PR3 |  |  |
| **34** | CREG1 | **68** | KIF3B | **102** | SEPT_6 |  |  |

**Supplementary table 5. Sequence of primers used in reverse transcription quantitative PCR (RT-qPCR)**

| **Gene symbols** | **Sequence** |
| --- | --- |
| EGR-1_Forward | AAA GTT TGC CAG GAG CGA TG |
| EGR1_Reverse | CAG GGG ATG GGT ATG AGG TG |
| GAS1_Forward | CCT CAT TCA GCT CAA CCA CA |
| GAS1_Reverse | CTT GGT GGA CTT GCA GTT CT |
| RAMP3_Forward | TGC AAC GAG ACA GGG ATG C |
| RAMP3_Reverse | GCA TCA TGT CAG CGA AGG C |
| CXCR7_Forward | ACA GGC TAT GAC ACG CAC TG |
| CXCR7_Reverse | ACG AGA CTG ACC ACC CAG AC |
| CD59_Forward | ATT TCA ACG ACG TCA CAA CCC |
| CD59_Reverse | CAG AAA TGG AGT CAC CAG CAG |
| GAPDH_Forward | ATC ATC AGC AAT GCC TCC T |
| GAPDH_Reverse | CAT CAC GCC ACA GTT TCC |

**Supplementary Figure 1. ROC curve of EGR1 expression and their area under the curve (AUC)**


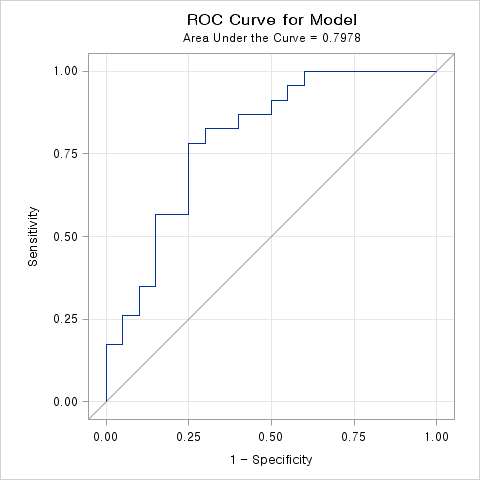

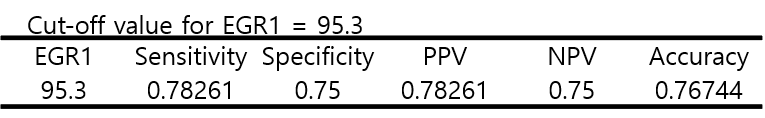


**Supplementary Figure 2. Kaplan-Meier survival curves for ENKTL patients according to EGR1 expression**


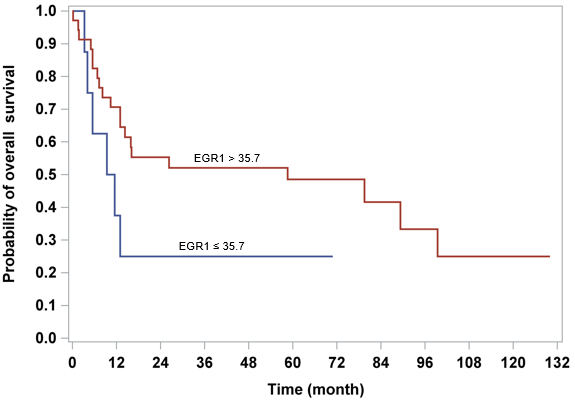


Cut-off value for EGR1 = 35.7

**Supplementary Figure 3. Knockdown efficiency of siRNA targeting CD59, CXCR7, GAS1 and RAMP3 in NK92MI cells**

**Supplementary Figure 4. Apoptosis analysis through cleaved PARP and caspase-3**


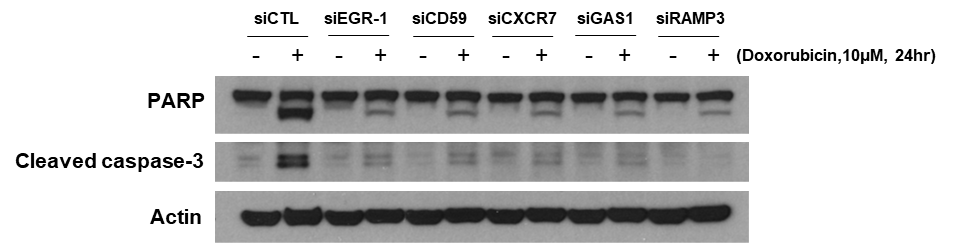

Supplement: Supplementary file 1 — Supplementary Information. [file 41598_2021_89754_MOESM1_ESM.docx]
